# 

**Published:** 1919-11-08

**Authors:** 


### Buildings Contemplated and in Progress.

CHIRK.—A strong representative Committee has been formed with the object of raising £5,000 for a proposed hospital for Chirk, Denbighshire.

COLWYN BAY.—The war memorial is partly to take the form of the extension of the present hospital accommodation at a cost, for fifty to sixty beds, of £50,000.

DEPTFORD.—The War Memorial Committee has decided upon the provision of a children's ward in the Miller Hospital. It is estimated that the cost will be £7,000.

GRAYSHOTT.—Efforts are being made to raise £10,000 for providing a joint cottage hospital for Grayshott, Headley, and Hindley as a war memorial. A site at Grayshott has been given.

HELSTON.—It is proposed to erect a new cottage hospital for Helston and district.

PORTSMOUTH.—The Town Council has received sanction from the Ministry of Health to borrow £1,876 for a new smallpox hospital.

### Hospital and Institutional Results.

**St. Mary's Hospital, Paddington.**—Although legacies yielded in 1918 the abnormally high sum of £9,157, and £8,077 was received from the Government for the treatment of wounded soldiers, the whole of these moneys were swallowed up by the necessary expenditure of the year, and still left a deficiency of £4,515. During the war annual subscriptions have fallen off by nearly £1,000.

**Metropolitan Hospital.**—The average cost per occupied bed was last year only £2 4s. 9d. a week. This is a very low figure in these expensive times.

**West London Hospital.**—With a total of 168 available beds it is good to get an average occupation of 160. The average cost per week in 1918 was £2 11s. 6d. There was a balance of expenditure over income of £3,306.

**Bradford Royal Infirmary.** The financial report is disquieting reading. The balance due to the bank has increased from £1,370 to £6,698. Legacies which, provincial fashion, do not appear in the income and expenditure account, amounted to £577, and there was a donation of £100 for endowment. One bright feature is that annual subscriptions increased from £7,542 in 1917 to £8,743 in 1918. This meant hard work on the part of somebody.

**Edinburgh Royal Infirmary.**—The financial year runs from October to October. Why? The annual subscription list is increased by nearly £4,000, and expenses are £10,000 over 1917. Legacies and donations over £100 amounted to £40,555.

**Royal Devon and Exeter Hospital.**—The hospital has a successful organisation for educating young patients during their stay in the wards. From the report of the chaplain it is evident that the efforts of the teachers are highly appreciated.

**Royal Southern Hospital, Liverpool.** — What the hospital gained in 1917, when there was a surplus of £8,541, it partly lost in 1918 through the deficit of £4,398. The hospital asks for increased endowment and money to provide better accommodation for the nursing staff.

**Royal Victoria Infirmary, Newcastle-on-Tyne.**—A school of massage and medical gymnastics was opened late in 1918 with Miss M. G. Nicholson (Teacher's Certificate, I.S.T.M.) as the head. Workmen's contributions to the infirmary were £33,300, exceeding 1917 by £3,000.

**Chelsea Hospital for Women.**—The hospital finished the year with a balance on the right side of £2,305. The War Office paid £4,571 for the cost of the officers' wards.

**Queen Charlotte's Lying-in Hospital.**—The hospital is usefully developing in the direction of pre-natal and infant-welfare work. One thousand six hundred and nineteen married women were delivered in their own homes by the hospital midwives.

**Royal London Ophthalmic Hospital.**—There were 98,186 attendances of out-patients last year. In an eye hospital each attendance means something more than a brief interview with a doctor, and the large figure quoted shows that the labours of the old "Moorfields" Hospital are very extensive.

**The Royal Maternity Charity.**—The charity has helped 570,502 cases since its foundation in 1757; 369 mothers between the ages of nineteen and forty-nine were attended last year. The cost per patient was £3 17s. 8d. There were twelve cases of twins and one of triplets.

**The South London Hospital for Women.**—The income and expenditure account shows a balance on the wrong side of £1,439. The average weekly cost of each in-patient was £2 15s. 8d. An appeal is made for empty bottles in order to reduce the dispensary cost.

### PASSING EVENTS AND TOPICS.

### Gift to Red Cross Worker.

THE miners of Notts and Derbyshire and other working men of the neighbourhood have presented a piece of silver plate to Sir H. Dennis Bayley, of Nottingham, who was instrumental in raising £387,000 for the Red Cross, to which the miners contributed £54,000.

### The Royal Sanitary Institute.

IN connection with the thirty-first Congress of the Institute a Health Exhibition will be held in Birmingham at Bingley Hall from July 17 to August 17, 1920. The meeting will be attended by a large number of official delegates from local authorities, delegates from Colonial and foreign Governments, as well as architects and engineers, members of the Institute.

### Alexandra Day.

THE collection in London and the suburbs on Alexandra Day amounted to £30,048. After payment of the cost of the flowers and other expenses, over £25,000 remained to be distributed amongst London charities, including hospitals. The Committee dealt with the awards at a recent meeting at the Mansion House, when the Lord Mayor presided. Queen Alexandra, at the Committee's wish, recommended grants to various charities, and the borough Mayors and others in authority in various districts were asked to nominate others.

### The Black Prince Cot.

QUEEN'S HOSPITAL FOR CHILDREN, Hackney Road, was the scene recently of an interesting ceremony, when a cot, endowed by the subscriptions of relatives and friends of the officers who went down in His Majesty's ship *Black Prince*, was dedicated by Archdeacon Ingles, Chaplain-General of the Navy, and unveiled by Admiral F. J. Fremantle. The inscription plate, in the centre of which is a photograph of the vessel, bears the words: "H.M.S. *Black Prince*. This cot, supported by the ship's company, was endowed in 1918-19 in perpetuity to the glorious and undying memory of the officers and men of H.M.S. *Black Prince*, sunk in the battle of Jutland, May 31, 1916." Then follow the names of the captain, commander, and other officers.

### The Great Northern Central Hospital.

THE PRINCE OF WALES will preside at a Festival Dinner at the Conaught Rooms on December 12, in connection with the Great Northern Hospital and its Islington War Memorial scheme. A Masonic service was held at the Parish Church of St. Mary, Islington, on November 2, at 3 P.M.

### Rationing in Institutions.

A CIRCULAR of Revised Procedure for Residential Establishments, Catering Establishments, and Institutions (R.R. 8) has been issued. One important direction is that all in-patients must hand their cards on entering to the hospital authorities, and those who do not are to be reported to the Food Office. A new Memorandum (M.G.N.R. 84) as to Cream and Extra Rations for Invalids has also been issued.

### A Veteran Chaplain.

AFTER some thirty-five years of service, the Rev. J. C. Crawford has resigned the position of Chaplain to the Cane Hill Mental Hospital. Mr. Crawford, who graduated M.A.(Oxon.), was ordained deacon in 1875 and priest in 1878, being appointed eight years later as Chaplain to the County Asylum.

### Medical Appointments.

At the General Hospital, Birmingham, the following appointments have been made: Mr. J. B. Leather, F.R.C.S.E., and Mr. B. T. Rose, F.R.C.S.E., assistant surgeons, and Mr. W. A. Clements surgical registrar. The last is a new appointment, created in connection with an improved service in the out-patient and casualty department.

### King's College Hospital.

Medical and Surgical Appointments.

*Junior Physician*: A. C. D. Firth, M.A., M.D., B.C. (Cantab), M.R.C.P. (Lond.), M.R.C.S. (Eng.), Hon. Capt. R.A.M.C.

*Senior Urologist in charge of Urological Department*: I. W. Thomson Walker, F.R.C.S. (Eng.), M.B., C.M. (Edin.), M.R.C.S., L.R.C.P. *Junior Urologist and Junior Surgeon*: John Everidge, O.B.E., F.R.C.S. (Eng.), M.R.C.S., L.R.C.P. *Senior Orthopædist*: Harold A. T. Fairbank, F.R.C.S. (Eng.), M.B., M.S. (Lond.), M.R.C.S., L.R.C.P. *Junior Orthopædist and Junior Surgeon*: C. Jennings Marshall, M.S., M.D. (Lond.), F.R.C.S. (Eng.). *Junior Neurologist*: S. A. Kinnier-Wilson, M.A., M.B., Ch.B. (Edin.), B.Sc., M.D., M.R.C.P. F.R.C.P. (Lond.).

New Special Departments.—The following new special departments have been established as forming part of the scheme of the construction: 1. Cardiology. 2. Therapeutics and Applied Pharmacology. 3. Neurology in connection with Psychiatry Out-patient Department. 4. Genito-Urinary with Urological Department. 5. Orthopædics and Fractures.

### Matrons' and Sisters' Appointments.

University College Hospital Medical School.—The two Goldsmid Entrance Exhibitions, offered annually for competition in September, have been awarded this year to Mr. J. H. Porter, Cambridge University, and Miss E. M. Turner, Cambridge University.

The Gloucestershire Royal Infirmary and Eye Institution.—Miss Margaret Ripley has been appointed matron. She was trained at Guy's Hospital, London, where she was afterwards sister. She has done military nursing in France, and since her return to England has been matron, Hospital for Officers, Swansea.

Cottage Hospital, Colwyn Bay.—Miss Margaret Evans has been appointed nurse-matron. She was trained at the Royal Infirmary, Edinburgh, and has since held appointments at Queen Mary's Military Hospital, Whalley, Union War Hospital, Southampton, Leeds Hospital for Women and Children. She has also been charge sister and night superintendent at the Military Hospital, Ripon.

Kent County Ophthalmic Hospital, Maidstone.—Miss D. M. Milton has been appointed matron. She was trained at the Leeds General Infirmary, where she became sister and sister-housekeeper. She has been theatre sister, Leicester Royal Infirmary, and has done war service in the R.N.N.S.R.

County Hospital, Hertford.—Miss Curragh has been appointed sister. She was trained at the South Devon and East Cornwall Hospital, Plymouth, and has since been sister, Hospital for Women, Soho, London, and night sister Royal National Hospital, Ventnor.

Royal Hospital, Chesterfield.—Miss W. Sutcliffe has been appointed theatre sister. She was trained at the Royal Infirmary, Sheffield, where she was theatre staff nurse and temporary sister.

Essex County Hospital, Colchester.—Miss J. Whittam has been appointed sister of the out-patients' and massage department. She was trained at Guy's Hospital, and has since been ward and night sister Essex County Hospital, assistant matron Taunton General Hospital, sister Massage and Electrical Department, T.F.N.S., Plymouth, and theatre sister 57th General Hospital, France. She holds the certificate of the I.S.T.M.

### The College of Nursing (Limited by Guarantee).

### LONDON CENTRE.

Thursday, November 13, 8 p.m. in the Rooms of the Medical Society, Chandos Street.—Lecture by Miss Christie: "Factors in Determining Prices. Miss Christie's first lecture last week was well attended and much enjoyed.

### LIVERPOOL CENTRE.

A meeting for members of the College of Nursing, Liverpool Centre, was held on Monday, October 27, at the Royal Infirmary, Liverpool, Miss Cummins, R.R.C., in the chair. Miss Cowlin, Organising Secretary of the College of Nursing, spoke on "The Future of the Nurse." The address, both by reason of the subject and because of its manner of handling, made a considerable impression on the audience.

### EDINBURGH CENTRE.

Wednesday, November 19, at 3.30.—Annual Meeting at the Edinburgh Café, 77 Princes Street. Members are reminded that subscriptions for the ensuing year are then due.

### Gifts and Bequests.

Mr. W. H. Place, of Darwen, Lancashire, left £250 to the Darwen and District Nursing Association.

Mrs. Sarah Arbuckle, of Kilmarnock, has bequeathed £1,000 to the Kilmarnock Infirmary and £350 to the Kilmarnock Nursing Association.

Mr. Robert Anderson, of Glasgow, has left £1,000 each to the Glasgow Royal Infirmary, the Western Infirmary, the Glasgow Samaritan Hospital for Women, and the Royal Hospital for Sick Children, and £500 each to the Victoria Infirmary and the Orphan Homes of Scotland.

Of her shares in the Dublin Artisans' Company the Countess of Meath left, after her husband's death, 500 to the Hospital for Jews at Jerusalem, 1,000 to the Meath Home of Comfort, and 800 to the Brabazon Home of Comfort, Reigate. The reversionary residue is left to miscellaneous charitable objects at the discretion of the executors.

Mr. John Huxtable, of Barnstaple, has left £100 to the Barnstaple and North Devon Infirmary.

Mr. Henry Stephenson, of Lytham, Lancs, has bequeathed £500 to the Victoria Hospital, Accrington.

The Great Northern Central Hospital has received from the League of the Roses £250, making £950 since January.

Lord Yarborough has given £1,000 towards the purchase of Nunns Farm as a site for the War Memorial Hospital at Grimsby.

Reigate and Redhill Hospital benefits to the extent of £100 under the will of the late Mr. Frederick Gregory, of Earlswood, Surrey.

The Crewe Memorial Cottage Hospital has received a cheque for £300 from the London and North-Western Railway Employees' Committee, Crewe, on behalf of the hospital funds.

